# Sarcopenic Obesity and Cardiovascular Disease: An Overlooked but High-Risk Syndrome

**DOI:** 10.1007/s13679-024-00571-2

**Published:** 2024-05-16

**Authors:** Saeid Mirzai, Salvatore Carbone, John A. Batsis, Stephen B. Kritchevsky, Dalane W. Kitzman, Michael D. Shapiro

**Affiliations:** 1 Section on Cardiovascular Medicine, Department of Internal Medicine, Wake Forest University School of Medicine, Winston-Salem, NC USA; 2 Pauley Heart Center, Division of Cardiology, Department of Internal Medicine, Virginia Commonwealth University, Richmond, VA USA; 3 Department of Kinesiology & Health Sciences, College of Humanities & Sciences, Virginia Commonwealth University, Richmond, VA USA; 4 Division of Geriatric Medicine, School of Medicine, University of North Carolina at Chapel Hill, Chapel Hill, NC USA; 5 Department of Nutrition, The Gillings School of Global Public Health, University of North Carolina at Chapel Hill, Chapel Hill, NC USA; 6 Section on Gerontology and Geriatric Medicine, Department of Internal Medicine, Wake Forest University School of Medicine, Winston-Salem, NC USA; 7 Center for Prevention of Cardiovascular Disease, Medical Center Blvd, Wake Forest University School of Medicine, Winston-Salem, NC 27157 USA

**Keywords:** Aging, Sarcopenia, Obesity, Sarcopenic obesity, Risk factors, Cardiovascular disease

## Abstract

**Purpose of Review:**

Sarcopenic obesity (SO), defined as the coexistence of excess fat mass and reduced skeletal muscle mass and strength, has emerged as an important cardiovascular risk factor, particularly in older adults. This review summarizes recent findings on the diagnosis, prevalence, health impacts, and treatment of SO.

**Recent Findings:**

Growing evidence suggests SO exacerbates cardiometabolic risk and adverse health outcomes beyond either condition alone; however, the heterogeneity in diagnostic criteria and the observational nature of most studies prohibit the evaluation of a causal relationship. This is concerning given that SO is increasing with the aging population, although that is also difficult to assess accurately given wide-ranging prevalence estimates. A recent consensus definition proposed by the European Society for Clinical Nutrition and Metabolism and the European Association for the Study of Obesity provides a framework of standardized criteria to diagnose SO.

**Summary:**

Adopting uniform diagnostic criteria for SO will enable more accurate characterization of prevalence and cardiometabolic risk moving forward. Although current management revolves around diet for weight loss coupled with resistance training to mitigate further muscle loss, emerging pharmacologic therapies have shown promising results. As the global population ages, diagnosing and managing SO will become imperative to alleviate the cardiovascular burden.

## Introduction

In an era marked by a global obesity epidemic and an aging population, the interplay of obesity and age-related changes has emerged as a critical public health concern. Obesity is widely recognized as a significant risk factor for cardiometabolic diseases, imposing a substantial burden on quality of life, disability, and life expectancy [1]. Simultaneously, there is a demographic shift, with adults 65 and older making up 9% of the global population in 2019, projected to rise to 21% by 2050 [2]. As individuals age, they undergo significant physiological changes, namely, fat mass gain and redistribution, skeletal muscle mass loss, and reduced muscular strength [3]. These age-related changes are intertwined with cardiovascular disease (CVD), such as atherosclerosis and heart failure (HF), sharing inflammatory, metabolic, and hormonal determinants [3]. Further exacerbating this process is the higher frequency of other comorbidities, such as diabetes and a sedentary lifestyle, in older adults. These complex factors highlight the intricate connection between the aging process, the muscle-fat interplay, and their relationship with CVD.

Growing evidence suggests that obesity and sarcopenia independently contribute to increased CVD risk [4, 5]. This leads one to expect a high CVD risk in older adults with sarcopenic obesity (SO), a syndrome characterized by the coexistence of sarcopenia and obesity. Yet, interestingly, amidst the conventional understanding of obesity as a CVD risk factor, an enigma persists—the so-called obesity paradox [6]. Some studies have suggested the counterintuitive notion that older individuals with established CVD classified as overweight or with obesity have better prognoses [6]. This paradox raises the intriguing question of whether the presence of obesity mitigates the negative impact of sarcopenia.

In this review, we describe the current literature focusing on SO and its relationship with CVD risk factors, CVD, and mortality, seeking to unravel the complex mechanisms and clinical implications of this compelling interplay. The discussion focuses on the findings and limitations of pooled analyses and recent studies not included within them. Due to the narrative nature of this review, the Preferred Reporting Items for Systematic Review and Meta-Analysis protocols are not strictly adhered to. The literature retrieval process focused on key terms related to the covered topics, with the most rigorous search conducted for the clinical data section.

## Pathophysiology

**Fig. 1:**
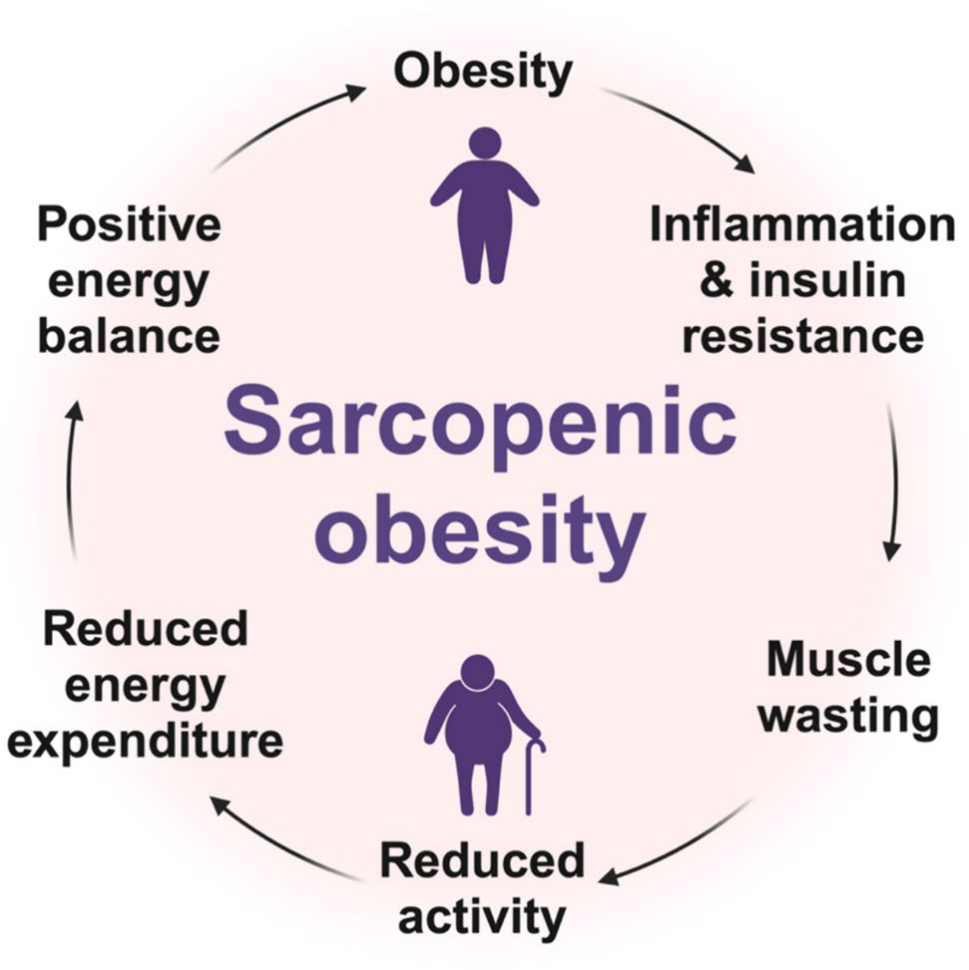
Simplified diagram of the self-perpetuating cycle linking obesity with muscle wasting

Aging is associated with a gradual decline in muscle mass and function, known as sarcopenia, and an increase in fat mass with redistribution to metabolically deleterious depots [7]. These shifts in body composition make older individuals more susceptible to unfavorable metabolic changes, which can increase the risk for CVD [8]. Furthermore, while a sedentary lifestyle is a risk factor for sarcopenia, sarcopenia can exacerbate the age-related decrease in physical activity and basal metabolic rate, thereby promoting further muscle loss and fat gain [9]. Consequently, the gain in fat mass with altered distribution, characterized by reduced subcutaneous fat and increased visceral and ectopic (e.g., muscle and liver) fat, exacerbates changes such as inflammation and insulin resistance, worsening muscle loss and creating a self-perpetuating cycle (Fig. 1) [10, 11]. Sex-specific hormonal changes, such as decreased estrogen levels in postmenopausal women and declining testosterone levels in men, also play a role in these changes [12].

The pathophysiology of SO is complex and multifactorial, involving metabolic, endocrine, hormonal, and neuromuscular changes that collectively contribute to impaired muscle protein synthesis, increased muscle breakdown, and progressive loss of muscle mass and function; additional factors include reduced satellite cell function, higher burden of chronic diseases, chronic low-grade inflammation, oxidative stress, and mitochondrial dysfunction [13–21]. Sarcopenic obesity shares common etiological mechanisms with CVD, including the imbalance between pro-inflammatory adipokines and anti-inflammatory myokines, oxidative stress, and mitochondrial dysfunction [7, 22]. These factors can contribute to insulin resistance, hyperglycemia, and hyperinsulinemia, which can lead to vascular remodeling, endothelial dysfunction, and hypertension [23]. Subsequently, chronic CVD, like HF and coronary artery disease (CAD), can exacerbate muscle wasting [24–26]. The intricate interplay of these mechanisms underscores the complex pathogenesis of SO and its association with CVD.

## Diagnosis

**Fig. 2:**
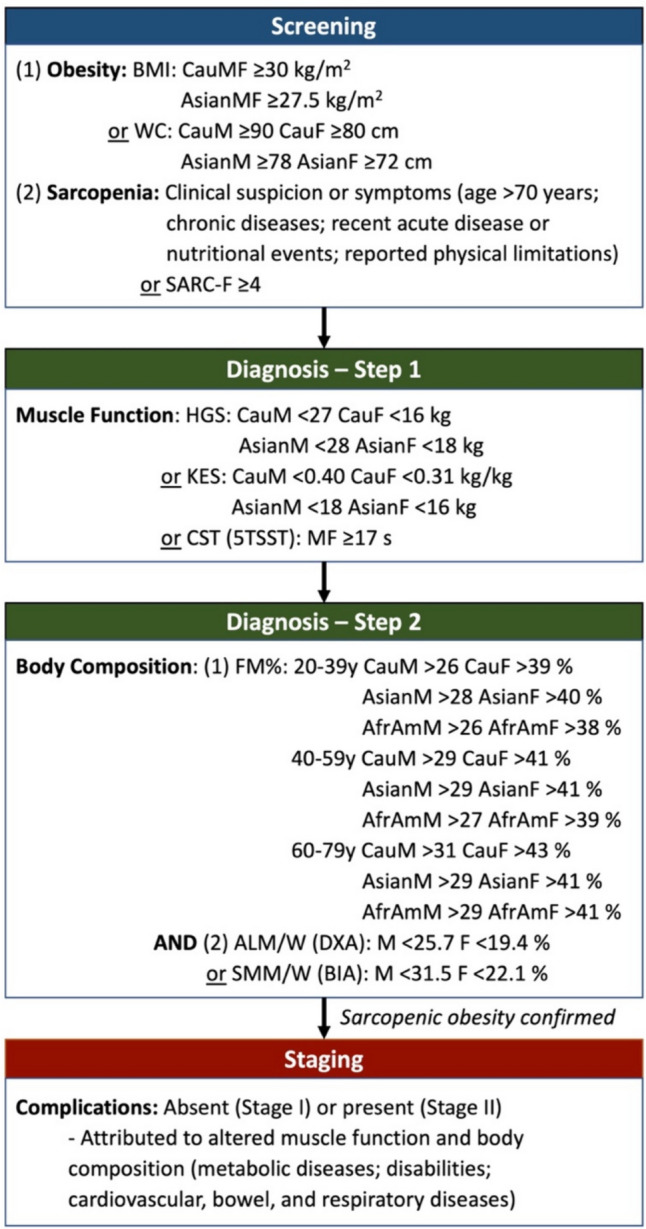
Diagnostic procedure and cutoffs for the assessment of sarcopenic obesity. Based on information from the ESPEN/EASO consensus definition [26]. BMI, body mass index; Cau, Caucasian; M, male; F, female; WC, waist circumference; SARC-F, strength, assistance with walking, rising from a chair, climbing stairs and falls; HGS, handgrip strength; KES, knee-extension strength; CST, chair stand test; STSST, 5-times Sit- to-Stand Chair test; FM%, fat mass percentage; AfrAm, African-American; ALM/W, appendicular lean mass adjusted to body weight; DXA, dual X-ray absorptiometry; SMM/W, total skeletal muscle mass adjusted by weight; BIA, bioelectrical impedance analysis

The first consensus statement for SO by an international expert panel was released in 2022 by the Sarcopenic Obesity Global Leadership Initiative, launched by the European Society for Clinical Nutrition and Metabolism (ESPEN) and the European Association for the Study of Obesity (EASO) [27••, 28]. In it, the expert panel proposes diagnostic criteria starting with screening for obesity using body mass index (BMI) or waist circumference (WC) by ethnic group-specific cutoffs, and sarcopenia by using surrogate indicators, such as risk factors, symptoms, or validated questionnaires (Fig. 2) [27••]. The diagnosis of SO is meant to be subsequently confirmed by assessing muscle weakness, followed by demonstrating evidence of altered body composition (increased fat mass and reduced lean mass). The consortium agreed that the latter could be measured using dual X-ray absorptiometry (DXA; preferred) or bioelectrical impedance analysis (BIA) [27••]. It is worth noting that lean mass from DXA or BIA is a surrogate of skeletal muscle mass, incorporating water content and being subject to fluctuations with volume status. The panel acknowledged the limitations of these methods versus computed tomography (CT), magnetic resonance imaging (MRI), and D3-creatine dilution but agreed that they adequately balance precision, accuracy, and availability.

The statement emphasized the importance of including muscle function, given its better prediction of health outcomes compared to muscle mass and frequent discrepancies between the two measures [29, 30•, 31]. Such discrepancies have been attributed to a lack of specificity of DXA or BIA measurements for skeletal muscle and their inability to capture muscle quality, composition, or neuromuscular impairment [27••]. The decision for muscle function to precede body composition was pragmatic, given the simplicity and availability of such measures compared to body composition analysis, particularly in clinical care (Fig. 2). Furthermore, the panel preferred assessment methods that focused on strength over performance measures such as gait speed to avoid potential clinical confounders (e.g., osteoarthritis in obesity); they recommended maximal strength testing between two limbs for handgrip or knee extensor strength without normalization for body mass [27••].

Following body composition analysis, the muscle mass measurements are normalized to body mass to account for the impact of higher muscle workload needed for daily activity in obesity [27••, 32]. Sarcopenia and obesity are diagnosed as distinct phenotypic traits rather than with integrated indices since clinical data do not currently support those. Obesity is characterized by an increased percentage of total body weight as fat mass, while sarcopenia is defined by a relative reduction in skeletal muscle mass when adjusted for body weight. The panel prefers appendicular lean mass (sum of the lean mass of the extremities) adjusted to body weight (ALM/W) by DXA; as alternatives, ALM/W or total skeletal muscle mass adjusted by weight (SMM/W) by BIA may be used (Fig. 2) [27••]. Although the group proposes consensus cutoffs, they acknowledge the need for further age, sex, and ethnicity-specific cutoffs [27••].

Finally, SO can be staged based on the absence (stage I) or presence (stage II) of at least one complication that can be attributed to physical dysfunction and altered body composition (Fig. 2) [27••]. The staging aims to stratify patients based on clinical severity and higher risk for poor outcomes who need more aggressive treatment and follow-up. The use of the ESPEN/EASO consensus definition has been limited in the literature, given its recent publication, but emerging studies have confirmed its validity for identifying SO and predicting poor outcomes [33–35]. Specifically, compared to the European Working Group on Sarcopenia in Older People (EWGSOP2) sarcopenia criteria, the ESPEN/EASO criteria has better identified SO among older men (≥ 70 years) and associated with higher muscle dysfunction, disability, and falls compared to those without SO [34].

## Epidemiology

It is difficult to accurately establish the prevalence of SO given the long-standing lack of a consensus definition (Table 1). For example, a 2020 systematic review of SO definitions among 75 studies from 2007 to 2018 found 19 and 10 different measures of sarcopenia and obesity, respectively, with various adjustments and cutoffs; few studies appropriately diagnosed sarcopenia as the coexistence of low muscle mass and function [36]. Furthermore, a 2013 study of eight definitions found 19- to 26-fold variations in sex-specific prevalence of SO in older adults ≥ 60 years in the 1999–2004 National Health and Nutrition Examination Surveys [37•].

**Table 1:** The various definitions used in the literature for sarcopenia, obesity, and sarcopenic obesity. From Liu et al. [38], with permission; © 2022 World Obesity Federation

**Sarcopenia (Study number = 82)**		**Obesity (Study number = 80)**		**Sarcopenic Obesity (Study number = 81)**	
ASM/ht^2^	36.14%	BF%	47.50%	Sarcopenia + Obesity	95.06%
(ASM or SMM)/weight	9.64%	BMI	31.25%		
ASM/BMI	3.61%	WC	8.75%		
ASM or SMM	4.82%	Visceral fat	2.50%	Linear regression model of appendicular fat-free mass	4.94%
(ASM or SMM)/(ht or ht^2^ or BMI) + HGS and/or functional test	36.14%	Combined	6.25%		
HGS	3.61%	Other	3.75%		
Other	6.02%				

*ASM* appendicular skeletal muscle mass, *BMI* body mass index, *SMM* skeletal muscle mass, *HGS* handgrip strength, *ht*^*2*^ height square, *SO* sarcopenic obesity

Based on the current literature, a 2021 meta-analysis assessing the global prevalence of SO in older adults ≥ 60 years using 50 studies (n = 86,285) from 2002 to 2020 demonstrated a pooled SO global prevalence of 11% (95%CI 10–13%) with high heterogeneity (I^2^ 99.5%) [38]. The definitions used greatly varied between studies (only 20% used both muscle mass and function to identify sarcopenia), with prevalence ranging from 0.1% to 48%. On subgroup analyses, the pooled prevalence of SO was higher in South America (21%, 95%CI 13–29%; eight studies) and North America (19%, 95%CI 10–27%; five studies), among those ≥ 75 years (23%, 95%CI 15–30%; three studies), and in hospital settings (16%, 95%CI 6–26%; two studies) compared to the community (11%, 95%CI 10–13%; 48 studies) [38].

Another 2023 meta-analysis of adults ≥ 50 years found a similar SO prevalence of 9% in both men and women across 48 studies from 2000 to 2020, but it differed by 15% when stratified by studies that adjusted muscle mass for weight as opposed to height^2^ (23% vs. 8%, respectively) [39••]. They also found South America (13%, 95%CI 8–18%) and Europe (12%, 95%CI 9–15%) to have the highest prevalence of SO across regions, though publication bias from unpublished low-prevalence cohort data may have limited the estimates [39••]. The established ESPEN/EASO criteria aim to overcome the major limitation of varying methodologies in the current literature. Following such a consensus algorithm will be necessary for future studies to estimate the prevalence of SO accurately.

## Clinical Data

### Sarcopenic Obesity and Cardiovascular Disease Risk Factors

Given the independent association of sarcopenia and obesity with metabolic disorders [40, 41], an additive association may be expected with SO. However, until now, cross-sectional studies have yielded inconsistent results. A 2023 meta-analysis by Liu et al. sought to study the relationship between SO and CVD risk factors among studies including adults ≥ 50 years [39••]. The authors found individuals with SO, compared to those without sarcopenia or obesity (reference group), to have a higher risk of prevalent hypertension (11 studies, n = 21,049; OR 1.99, 95%CI 1.34–2.97; I^2^ 87.5%), dyslipidemia (three studies, n = 4,123; OR 2.50, 95%CI 1.51–4.15; I^2^ 0.0%), diabetes (14 studies, n = 21,351; OR 2.02, 95%CI 1.39–2.93; I^2^ 82.6%), and metabolic syndrome (five studies, n = 6,079; OR 4.31, 95%CI 2.23–8.35; I^2^ 87.4%) [39••]. The risk of hypertension was slightly higher in obesity (OR 2.19, 95%CI 1.45–3.31) but non-significant in sarcopenia; risk of dyslipidemia was slightly higher in obesity (OR 2.68, 95%CI 1.10–6.51) but non-significant in sarcopenia; risk of diabetes was non-significant in obesity or sarcopenia; and risk of metabolic syndrome was similar in obesity (OR 4.31, 95%CI 2.23–8.35) but non-significant in sarcopenia [39••]. However, one needs to be cautious when interpreting the results of these combined analyses. The diverse diagnostic criteria used in the studies and their predominantly cross-sectional design preclude establishing a causal relationship, especially considering the potential for reverse causation. Also, the lack of consistent adjustment for major confounders, such as physical activity or cardiorespiratory fitness (CRF), introduces the potential for inaccurate conclusions.

Several studies, primarily focused on different age groups, were not included in the aforementioned meta-analysis but are worth mentioning. For example, hypertension has also been studied in younger cohorts. A 2013 cross-sectional analysis of 6,832 Korean participants (≥ 19 years) from the 2009 Korea National Health and Nutrition Examination Survey (KNHANES) categorizing participants by appendicular skeletal muscle mass adjusted to body weight (ASM/W) by DXA and WC found the highest risk of hypertension, compared to the reference, in SO (aOR 2.91, 95%CI 1.67–5.05) [42]. In contrast, a 2022 cross-sectional analysis of 4,021 Iranian participants (35–65 years) from the Ravansar Non-Communicable Disease cohort study (2014–2017) categorizing patients using handgrip strength (HGS), SMM by BIA, and WC found SO (OR 3.83, 95%CI 2.81–5.22), obesity, and sarcopenia to be associated with a higher risk of hypertension, in descending strength, compared to the reference; however, only obesity remained significant on adjusted models [43]. The contrasting findings between these studies underscore the importance of standardized diagnostic criteria for SO, as the variations might influence the observed associations. Furthermore, the generalizability of the findings may be limited by the geographic differences with potentially variable body composition, dietary, and activity patterns.

While the link between SO and dyslipidemia has often been unclear, a growing number of studies suggest a relationship. For example, significant associations between SO and dyslipidemia (CVD risk-based definition) were seen in a 2021 study using 2008–2011 KNHANES (17,546 adults; ≥ 19 years; aOR 1.53, 95%CI 1.19–1.98) and the Korean Genome and Epidemiology Study (5,126 adults; ≥ 40 years; aOR 1.35, 95%CI 1.15–1.58) where SO was defined by ASM/BMI by DXA and SMM/BMI by BIA, respectively, along with WC [44]. Additionally, a 2015 cross-sectional study using the Framingham risk score in 3,320 Korean adults (≥ 40 years) from the 2010 KNHANES found SO (ASM/weight by DXA and BMI) to have a significantly higher risk for 10-year CVD risk ≥ 20% compared to the reference (aOR 2.49, 95%CI 1.53–4.06 in men; aOR 1.87, 95%CI 1.02–3.41 in women); sarcopenia and obesity were not significantly different [45].

In the context of SO, diabetes is a particularly important comorbidity, given that obesity is a driver of diabetes, and diabetes has a bidirectional relationship with sarcopenia and frank skeletal muscle dysfunction; this relationship suggests that individuals with diabetes are at high risk for SO [46, 47]. A 2019 meta-analysis studied the risk for diabetes in adults with overweight and obesity across 11 studies (primarily cross-sectional; n = 60,118) and found SO (by various definitions) to be associated with an increased risk for diabetes (OR 1.38, 95%CI 1.27–1.50; I^2^ 60%) compared to the reference [48]. Furthermore, in a 2023 retrospective longitudinal study involving 36,304 Korean adults (≥ 20 years) who did not have diabetes, those with pre-SO (ASM/weight by BIA and WC) had the highest risk of developing diabetes [49]. On an adjusted model, compared to the reference, the risk was 1.57 times higher (95% CI 1.42–1.73) in pre-SO; pre-sarcopenia alone and abdominal obesity alone were also linked to a higher risk of developing diabetes [49]. Current hypotheses for the higher risk of diabetes in SO compared to the other phenotypes are the availability of less muscle for insulin-mediated glucose disposal, fatty infiltration of the muscles diminishing their insulin sensitivity, and the synergistic effect of the chronic inflammation caused by sarcopenia and obesity leading to worse insulin resistance and hyperglycemia [46]. However, it is essential to note the lack of consistent adjustment for visceral fat distribution and physical activity levels across the reported studies, which introduces the potential for confounding in the observed associations between SO and diabetes.

Beyond incidence and prevalence, SO can impact outcomes in patients with diabetes. A 2022 retrospective cohort study of 386 older Chinese participants (> 60 years) from the Ageing and Body Composition of Diabetes cohort found SO (HGS, gait speed, and ALM/height^2^ and BF% by DXA) to be significantly associated with a higher risk of all-cause death or fragility fracture (aHR 2.94, 95%CI 1.25–6.92) and incident composite CVD (aHR 6.02, 95%CI 1.56–23.15) [50]. A similarly increased risk of incident CVD was found in a 2018 study of 716 Japanese adults (> 20 years) with diabetes and SO diagnosed by DXA-based ASM/height^2^ and android to gynoid ratio (aHR 2.63, 95%CI 1.10–6.28) or android fat mass (aHR 2.57, 95%CI 1.01–6.54) but not BF% (aHR 1.67, 95%CI 0.69–4.02), indicating better risk prediction using abdominal fat, rather than total body fat, distribution [51]. There is also data to support an association between SO and accelerated chronic kidney disease development in patients with diabetes [52, 53].

Finally, recent studies have yielded inconsistent findings regarding the association between SO and metabolic syndrome. A 2020 meta-analysis of 12 studies (n = 11,308, ≥ 19 years, with overweight or obesity) found no significant difference in the risk of metabolic syndrome between individuals with SO and those without (RR 1.08, 95%CI 0.99–1.17; I^2^ 80.0%) [54]. This is despite the previously mentioned association between SO and metabolic syndrome risk in the pooled analysis of five studies by Liu et al. in 2023. However, it is important to note that Liu et al. applied more stringent exclusion criteria, excluding studies that considered SO a secondary outcome or included participants aged < 50 years [39••]. Also, it is interesting to note that a 2018 meta-analysis demonstrated associations between sarcopenia alone and metabolic syndrome, although sarcopenia and SO are distinct conditions with different clinical implications [55].

**Fig. 3:**
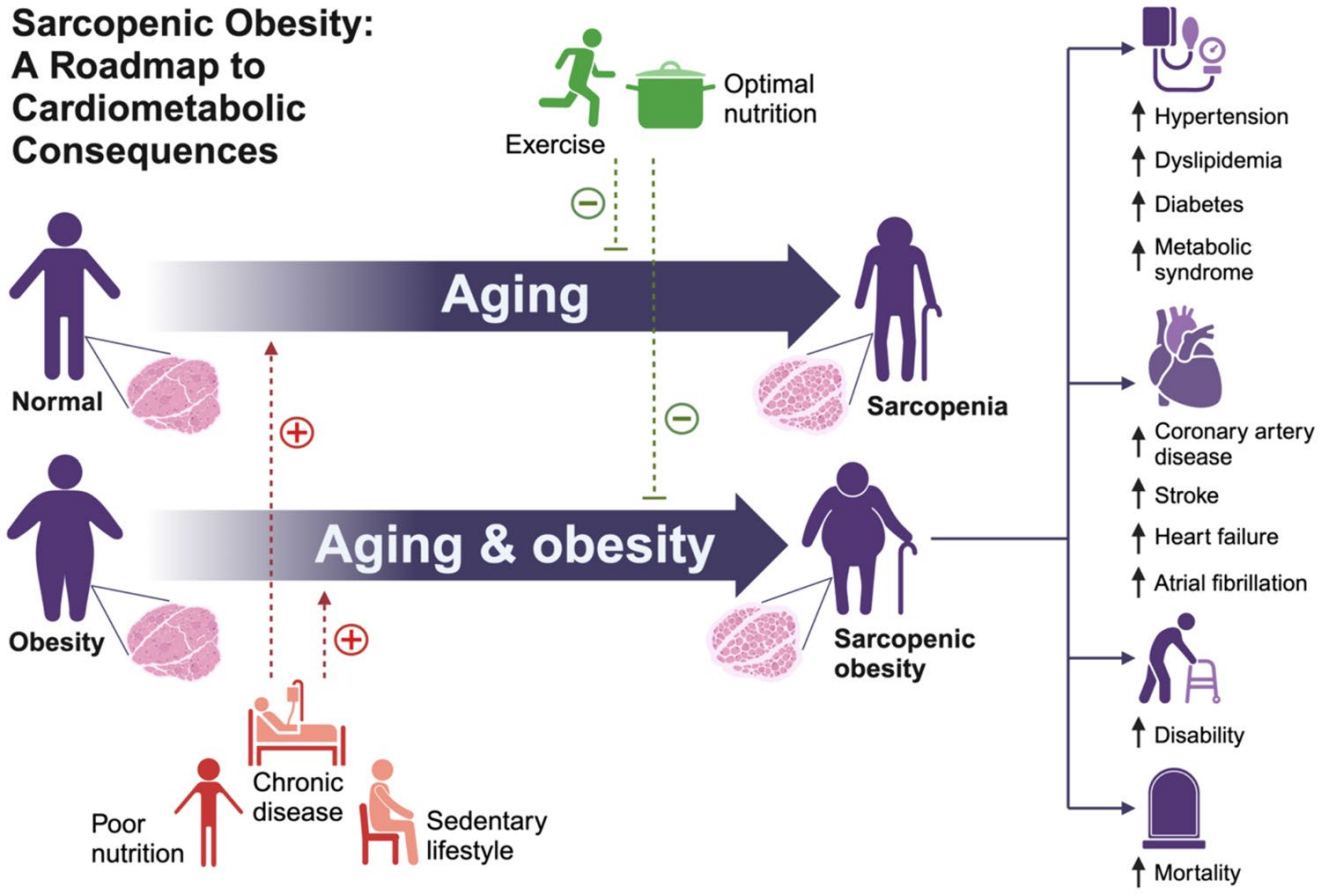
Gain of fat mass (obesity) and loss of skeletal muscle mass and function (sarcopenia) are expected changes with aging. However, individuals with obesity are at an increased risk for accelerated sarcopenia. Although the literature is limited and inconsistent, sarcopenic obesity has been associated with various cardiometabolic diseases, functional decline, and mortality. Greater focus on exercise, nutrition, and chronic disease management can help prevent or mitigate this often overlooked syndrome

In summary, the current evidence indicates an association between SO and the risk of hypertension, dyslipidemia, and diabetes, but less certain for metabolic syndrome (Fig. 3). The heterogeneous methodology likely drives the lack of association in some studies; therefore, further studies are needed to better identify associations between cardiometabolic risk factors and SO identified using the most current consensus definition with adequate control for potential confounding factors and participant follow-up to better establish causality.

### Sarcopenic Obesity and Cardiovascular Disease

The current cross-sectional and prospective literature studying the association between SO and the risk of CVD events and mortality has been limited and inconsistent, perhaps due to the different definitions of SO employed in these studies. Recently, Liu et al. quantitatively combined these studies in their 2023 meta-analysis [39••]. Pooled analyses of cross-sectional studies demonstrated that SO, compared to the reference, is associated with a higher risk for CVD events (five studies, n = 12,867; OR 1.97, 95%CI 1.25–3.11; I^2^ 62.8%), CAD events (two studies, n = 9,840; OR 2.48, 95%CI 1.85–3.31; I^2^ 0.0%), stroke (eight studies, n = 16,249; OR 1.82, 95%CI 1.47–2.26; I^2^ 0.0%), and other heart diseases (myocardial infarction [MI], angina pectoris, and HF) (11 studies, n = 21,071; OR 1.51, 95%CI 1.07–2.12; I^2^ 66.6%); significant associations of CVD and CAD events were not seen on pooling of two available longitudinal studies [39••]. Similarly significant associations between CVD events, CAD events, and other heart diseases were seen in the pooled analyses of sarcopenia but not obesity. For CVD-related death, data from three studies (n = 10,721) found the highest risk of death in those with SO (HR 1.63, 95%CI 1.01–2.62; I^2^ 75.2%) followed by sarcopenia (HR 1.38, 95%CI 1.19–1.60), but obesity did not significantly differ from the reference [39••].

The current meta-analyses have limitations given their utilization of studies with great heterogeneity across diagnostic criteria and mostly cross-sectional study design. A 2009 prospective cohort study, for example, compared the risk of incident CVD among 3,366 older US adults (≥ 65 years) from the Cardiovascular Health Study with SO defined by two definitions, one strength-based using HGS with WC and the other body composition-based using height-adjusted SMM by BIA with WC [56]. They found a stronger unadjusted association with CVD compared to the reference when SO was defined by muscle strength (HR 1.23, 95%CI 0.99–1.54) rather than mass (HR 1.10, 95%CI 0.81–1.48) [56]. Similarly, a 2014 study of older British adult men (n = 4,252, ≥ 60 years) from the British Regional Heart Study showed no significant association between SO (midarm circumference and WC) and CVD events or mortality [57], whereas a 2019 study of British adults (n = 452,931, 40–69 years) from the UK Biobank showed SO (HGS and BMI) to be associated with higher risk of CVD events (HR 1.42, 95%CI 1.31–1.55 in those without and HR 1.37, 95%CI 1.26–1.49 in those with CVD history) and CVD mortality (HR 1.78, 95%CI 1.45–2.18 in those without and HR 1.63, HR 1.36–1.95 in those with CVD history) [58]. These studies highlight the importance of functional testing in the SO diagnostic criteria.

Beyond the mentioned pooled analyses, past studies have also indicated a relationship between SO and atherosclerosis. A 2021 cross-sectional study of 19,728 Korean adults (≥ 20 years) showed pre-SO (ASM/weight by BIA and WC) to have the highest risk for coronary artery calcification (CAC) presence (score > 0; aOR 2.16, 95%CI 1.98–2.36) compared to the reference [59]. They also had higher total CAC incidence and progression (aHR 1.54, 95%CI 1.37–1.75) than the reference and pre-sarcopenia or obesity alone [59]. Similar results were found in a 2021 cross-sectional analysis of 1,282 Korean adults (mean age 58.1 years) where SO (ASM/weight by BIA and BMI) had a higher odds of high CAC score (score ≥ 100; aOR 1.92, 95%CI 1.16–3.18) compared to the reference [60]. It should be noted that the participants included in both studies had volunteered for health checkups and thus may not represent the general population.

The outcomes of clinically significant atherosclerotic events in individuals with SO remain inconclusive. A 2019 cross-sectional study of 99 hospitalized Brazilian adults (≥ 60 years) with acute MI found SO (HGS, gait speed, SMM/height^2^ by BIA, and abdominal circumference) was not associated with worse outcomes (inpatient complications, readmission, and length of stay) [61]. However, a 2021 retrospective cohort study of 303 hospitalized Japanese adults (median age 67 years) with ST-segment elevation MI found SO (ASM/height^2^ by DXA and abdominal visceral fat to subcutaneous fat ratio by abdominal CT) to have a significantly lower composite event-free survival rate compared to those without (composite outcomes of all-cause death, MI, ischemic stroke, hospitalization for HF, and unplanned revascularization), particularly in those below the median age (aHR 2.97, 95%CI 1.10–7.53) [62]. Variations in the selection criteria, diagnostic criteria, and the outcomes studied may account for the different results.

Excess adipose tissue, particularly visceral fat, and inflammation play key roles in the development and severity of HF with preserved ejection fraction (HFpEF) [63•]. Furthermore, poor exercise capacity, driven by skeletal muscle dysfunction, is a hallmark of the condition [64, 65•]. These suggest a relationship between SO and HFpEF [20, 64]. Notably, a 2017 cross-sectional study indicated an obesity-related phenotype of HFpEF (n = 99, mean age 65 years, BMI ≥ 35 kg/m^2^) associated with greater cardiac dysfunction, worse exercise capacity, and more hemodynamic derangements on exercise as compared to non-obesity HFpEF (n = 96, mean age 70 years, BMI < 30 kg/m^2^) and non-obesity non-HF reference group (n = 71, mean age 62 years, BMI < 30 kg/m^2^) [66]. Similarly, a 2019 secondary analysis of the Phosphodiesterase-5 Inhibition to Improve Clinical Status and Exercise Capacity in HFpEF (RELAX) trial indicated obesity-related HFpEF (n = 81, median age 64 years, BMI ≥ 35 kg/m^2^) to be associated with more severe signs and symptoms of HF with worse exercise capacity as compared to non-obesity HFpEF (n = 70, median age 73 years, BMI < 30 kg/m^2^) [67].

Beyond HFpEF, a 2021 cross-sectional study of 31,258 Korean adults (≥ 20 years) found that SO (ASM/weight by BIA and WC) had the greatest odds of left ventricular (LV) diastolic dysfunction (aOR 1.70, 95%CI 1.44–1.99) compared to the reference followed by obesity and sarcopenia; this remained significant on stratification by age at 65 years [68]. Similarly, another study of 733 Korean adults (20–79 years) showed that SO (SMM/weight by BIA and BMI) had the highest risk of LV diastolic dysfunction (aOR 4.27, 95% CI 2.41–7.57) compared to the reference followed by obesity and sarcopenia [69]. This study also demonstrated SO to have decreased exercise capacity compared with other phenotypes, similar to findings from a 2022 study of 40 adults (mean age 57 years) with symptomatic HF with reduced ejection fraction, which showed SO (SMM/height^2^ by BIA and BMI) to be associated with a clinically significant reduction in CRF compared to non-SO [69, 70]. Finally, a 2022 sub-analysis of 779 older adults (≥ 65 years) hospitalized for HF from the FRAGILE-HF study has indicated SO (HGS, gait speed, and ASM/height^2^ and FM% by BIA) as a risk factor for all-cause death (aHR 2.48, 95%CI 1.22–5.04) and lower physical function compared to the reference [71].

There are limited studies into the impact of SO on outcomes in electrophysiologic or structural diseases. A 2021 cross-sectional study of 2,432 Chinese adults (mean age 62.2 years) from the Shanghai Changfeng Study found sarcopenic overweight/obesity (ASM/height^2^ by DXA and BMI) to be associated with atrial fibrillation (aOR 5.68, 95%CI 1.34–24.12) [72]. Also, a 2016 retrospective cohort study of 460 older Canadian adults (mean age 81 years) found SO by pre-procedural CT (skeletal muscle and fat cross-sectional area at the third lumbar vertebra) to be associated with higher mortality post-transcatheter aortic valve implantation in sarcopenia, but not SO (HR 1.37, 95%CI 0.97–1.94) [73].

In summary, the current evidence indicates an association between SO and the risk of CVD (including CAD, stroke, HF, and atrial fibrillation), CVD events, and CVD mortality (Fig. 3). Further studies using consensus definitions will allow for better risk prediction, prevention, and targeted treatment of patients with SO.

### Sarcopenic Obesity and All-Cause Mortality

**Fig. 4:**
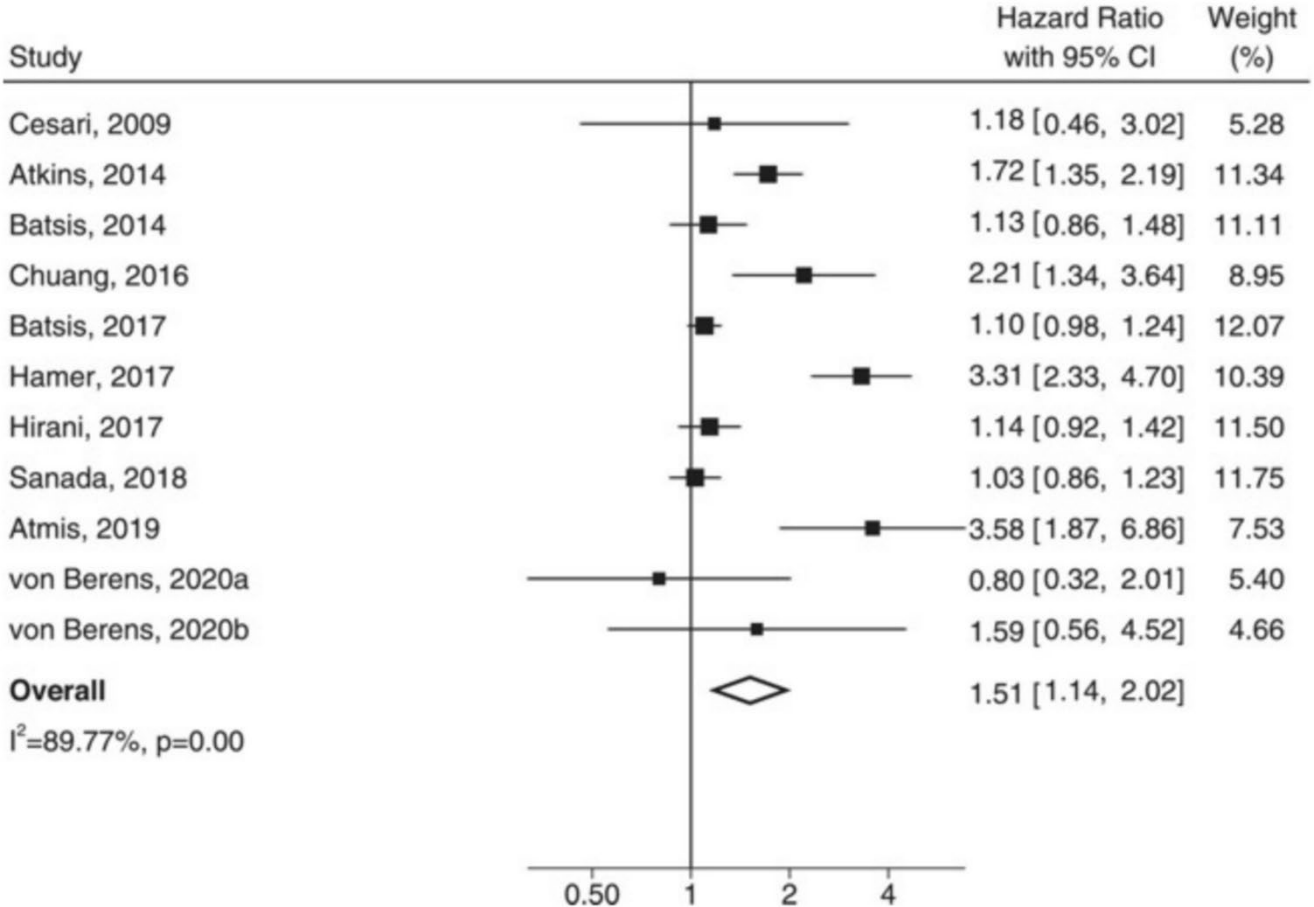
Random-effects meta-analysis showing the hazard ratio for all-cause mortality in patients with sarcopenic obesity. From Liu et al. [38], with permission; © 2022 World Obesity Federation

Beyond CVD and its risk factors, the 2023 meta-analysis by Liu et al. also studied all-cause mortality in SO [39••]. Their pooled analysis of 10 prospective cohort studies (n = 28,324, average age 64.6 to 79.5) with an average 9.6 follow-up years showed SO to have a 51% increased risk of all-cause mortality compared to the reference (HR 1.51, 95%CI 1.14–2.02; I^2^ 89.8%) (Fig. 4); a similar association was seen with sarcopenia (HR 1.49, 95%CI 1.27–1.75), but not obesity (HR 1.02, 95%CI 0.86–1.23) [39••]. Similarly, a 2019 meta-analysis by Zhang et al. studied the association of SO with all-cause mortality over a broad range of settings across 23 studies (n = 50,866, age 50–82.5 years) and found a significantly higher risk (HR 1.21, 95%CI 1.10–1.32; I^2^ 64.3%) compared to the reference [74]. The association was particularly high in hospitalized patients (HR 1.65, 95%CI 1.17–2.33; I^2^ 71.2%) compared to community-dwelling adults (HR 1.14, 95%CI 1.06–1.23; I^2^ 48.8%) [74]. Furthermore, a recent 2023 pooled analysis of 4,612 older adults (≥ 70 years) from three harmonized cohorts (Health 2000 Survey; Health, Aging and Body Composition Study; and Longitudinal Aging Study Amsterdam) showed probable sarcopenia with obesity (HGS and BMI or WC) to have a significantly higher risk of death compared to the reference (HR 1.36, 95%CI 1.13–1.64); probable sarcopenia-only had similarly higher risk, but the obese-only group risk did not differ from the reference [75]. Although the literature remains heterogeneous, further studies using the consensus definition will allow for better prognostication (Table 2).

**Table 2:** Future directions for sarcopenic obesity research. Based on information from the ESPEN/EASO consensus definition [26] and meeting proceedings [27••]

**Future directions**
• Use the current ESPEN/EASO consensus definition for greater homogeneity across the literature.
• Distinguish more clearly between the primary and secondary forms of the condition to devise treatment strategies tailored to each type.
• Explore the role of hormonal status (e.g., cortisol, testosterone, growth hormone/insulin-like growth factor-1 axis) on its pathogenesis and pathophysiology.
• Explore the cross-talk between bone, muscle, and adipose tissue related to age-related osteopenia-osteoporosis, sarcopenia, and overweight-obesity.
• Develop or validate age, sex, and ethnicity-based cut points for research and clinical practice consideration.
• Develop or validate screening methods, such as calf circumference.
• Confirm the reliability of using calf circumference, adjusted for body mass index, as an indicator of skeletal muscle mass.
• Compare the utility of different functional tests in various settings.
• Validate the use of different body composition adjustment methods for diagnosis.
• Study the prognostic ability of the proposed ESPEN/EASO consensus definition staging system.
• Increase the number and quality of randomized controlled trials studying the therapeutic effects of exercise, nutritional, pharmacologic, or combined interventions.
• Identify effective strategies for the mitigation of muscle loss during weight loss.

### The “Obesity Paradox”

Obesity, as defined by BMI, is a heterogeneous disease. Although BMI is a simple measure of obesity, individuals with similar BMI can have very different body compositions (e.g., muscle quantity/quality and fat quantity/distribution) and, thus, cardiometabolic risks. The “obesity paradox” refers to the paradoxical protective effect of being overweight or having mild obesity in some chronic diseases, particularly CVD; however, great controversy surrounds this concept [76•]. This is primarily due to significant methodological limitations with past literature supporting the “obesity paradox,” including the use of BMI without assessment of body composition phenotype, lack of consideration for CRF, reverse causation with antecedent weight loss from chronic disease elevating mortality risk, lack of consideration for weight loss from smoking, and lack of consideration for other confounders such as age or comorbidities [76•].

The studies presented in this review, along with a recent meta-analysis [39••], indicate the “obesity paradox” to not be present in SO, highlighting the importance of body composition analysis and phenotyping of obesity groups. For instance, given the association of higher muscle mass and subcutaneous fat quantity with better outcomes in past studies, one may argue that the absence of this paradox in SO is due to the loss of protective muscles or a potential tendency for these individuals to deposit fat in visceral rather than subcutaneous depots [77, 78]. Furthermore, beyond SO, despite past observational studies in middle-aged and older adults associating weight loss with increased mortality, a 2015 pooled analysis of 15 trials (1987–2013; n = 17,186) of intentional weight loss with lifestyle-based interventions in adults (average age 52 years) with obesity showed 15% lower all-cause mortality (RR 0.85, 95%CI 0.73–1.00; I^2^ 0) in participants randomized to weight loss compared to non-weight loss; this was significant on sub-group analysis of six trials with relatively older adults (≥ 55 years; RR 0.84, 95%CI 0.71–0.99) [79]. Overall, this evidence provides reassurance for the recommendation of weight loss in older adults with obesity despite past belief in an “obesity paradox,” but special consideration must be taken in SO, as discussed in the next section.

## Treatment

Lifestyle interventions, particularly physical activity, exercise training, and nutrition, are the cornerstone for preventing and managing SO [80]. Although few clinical trials have explicitly focused on SO treatment with uncertain results, weight loss trials in older adults with obesity have shown improvements in morbidity, mortality, and physical function [79–81]. Specifically, a 2011 clinical trial by Villareal et al. focused on older adults with obesity (n = 107, ≥ 65 years, BMI ≥ 30 kg/m^2^), comparing diet (weight loss by 500–750 kcal/day energy deficit with 1 g of high-quality protein/kilogram of body weight/day), exercise (aerobic and resistance training), diet-exercise, and no intervention, found the diet-exercise group to have the greatest improvement in physical function (e.g., Physical Performance Test score and peak oxygen consumption) compared to either intervention alone [82]. Similarly, a 2017 clinical trial by Villareal et al. compared diet-aerobic training (AT), diet-resistance training (RT), diet-AT-RT, and no intervention (no diet, AT, or RT) in older adults with obesity (n = 160, ≥ 65 years, BMI ≥ 30 kg/m^2^); the diet was like the 2011 study. They found diet-AT-RT to be most effective for improving physical function (e.g., Physical Performance Test score) [83••].

A long-standing concern with recommending weight loss with caloric restriction, with or without exercise, has been the general principle that one-fourth of weight loss is fat-free mass, with the remaining three-fourths being fat mass [84]; higher skeletal muscle losses have been reported in individuals with chronic disease, such as HFpEF (~ 35%) [85]. This is particularly concerning if weight cycling occurs where the regained weight is mostly fat rather than muscle [86]. However, studies investigating the effects of exercise on body composition in individuals with obesity have yielded promising results, including muscle gain with resistance exercise, fat loss and attenuated muscle loss with walking-type aerobic exercise, and fat loss and muscle gain through a combination of the two exercises [87, 88]. These findings are particularly relevant given the popularity of walking as a form of aerobic exercise among older adults, suggesting that it may be an effective strategy for achieving weight loss while preserving the muscles essential for ambulation and maintaining independence with advancing age [88].

Focusing on the combination of diet and exercise, the 2011 and 2017 trials by Villareal et al. evaluated changes in lean mass (by DXA), showing lower losses with diet-exercise compared to diet alone (-3% vs. -5%) and diet-AT-RT and diet-RT compared to diet-AT (-3% and -2% vs. -5%), respectively [82, 83••]. Similar comparisons were also made in a 2022 clinical trial by Brubaker et al. combining diet (weight loss by 300 kcal/day energy deficit with > 1.2 g of high-quality protein/kilogram body weight/day), RT, and AT versus diet-AT in older adults with obesity-related HFpEF (n = 88, ≥ 60 years, BMI ≥ 28 kg/m^2^). They found that both diet-RT-AT and diet-AT similarly improved exercise capacity and quality-of-life; however, diet-RT-AT also increased leg strength and muscle quality (ratio of knee extensor strength to thigh muscle area assessed by MRI) without attenuating skeletal muscle loss (by DXA) compared to diet-AT [89••]. This muscle loss raises concerns for the induction or exacerbation of SO in patients with obesity, particularly older adults with obesity and HFpEF; however, the improved muscle strength and quality despite the loss of mass may indicate preferential catabolism of low-quality muscle with overall enhanced quality.

Finally, although pharmacological therapies based on pathophysiology have been proposed (e.g., testosterone), current evidence does not support their use over lifestyle interventions [90]. However, emerging evidence has shown promising results for the benefit of glucagon-like peptide-1 receptor agonists, a class of agents approved for long-term weight management. For example, a 2021 clinical trial by Lundgren et al. compared exercise (mostly aerobic training), liraglutide (3.0 mg/day), combination, and placebo without exercise among adults with obesity without diabetes (n = 195, 18–65 years, BMI 32–43 kg/m^2^). They found that the combination strategy led to the greatest weight loss, body-fat percentage decrease (twice that of either intervention alone), and CRF improvement in addition to preserving lean mass (by DXA) [91••]. Similarly, the recent 2023 Effect of Semaglutide 2.4 mg Once Weekly on Function and Symptoms in Subjects with Obesity-related HFpEF (STEP-HFpEF) trial (n = 529, ≥ 18 years, BMI ≥ 30 kg/m^2^) showed semaglutide to be associated with greater reductions in HF symptoms and physical limitations, exercise function improvement, and weight loss than placebo [92••]. Further studies exploring the impact of pharmacologic agents, possibly coupled with diet and exercise, on body composition, CVD events, and survival in patients with SO will provide invaluable evidence for refining our strategies for tackling this complex condition.

## Conclusion

Sarcopenic obesity, characterized by the coexistence of sarcopenia and obesity, is an emerging risk factor for CVD. While research has revealed shared pathophysiological mechanisms between the two conditions, the literature remains heterogeneous in defining SO, making it difficult to characterize its true prevalence and association with cardiovascular outcomes. Moving forward, adopting the recent ESPEN/EASO consensus definition for SO will allow for building a more homogeneous evidence base to elucidate the impact of this syndrome and undertake clinical trials focused on its treatment. Current clinical management revolves around diet for weight loss coupled with resistance training to mitigate further muscle loss. Emerging pharmacologic therapies have shown promising results, but further research is needed to elucidate their impact on body composition when coupled with exercise. Given the rising public health burden, optimizing treatment strategies for SO may provide an opportunity to alleviate cardiovascular risk in an aging population.
